# Combination of SDF-1 and bFGF promotes bone marrow stem cell-mediated periodontal ligament regeneration

**DOI:** 10.1042/BSR20190785

**Published:** 2019-12-17

**Authors:** Mengting Xu, Xing Wei, Jie Fang, Li Xiao

**Affiliations:** 1 State Key Laboratory of Oral Diseases, National Clinical Research Center for Oral Diseases, West China Hospital of Stomatology, Sichuan University, Chengdu, China; 2 Department of Orthodontics, Stomatological Center, Shenzhen People’s Hospital, The Second Clinical Medical College of Jinan University, Shenzhen, Guangdong; 3 Department of Stomatology, Sichuan Academy of Medical Sciences and Sichuan Provincial People’s Hospital, Sichuan, China

**Keywords:** beagle dog, bFGF, BMSCs, periodontal ligament regeneration, SDF-1

## Abstract

Stromal cell derived factor-1 (SDF-1) and basic fibroblast growth factor (bFGF) were reported to induce the differentiation of bone marrow stem cells (BMSCs) into cells with characteristics of periodontal ligament fibroblasts. Thus SDF-1 and bFGF may play a positive role in BMSCs-mediated periodontal ligament regeneration. Here, the methylthiazolyldiphenyl tetrazolium bromide (MTT) assay was used to investigate the effect of scaffolds, SDF-1 and bFGF on BMSCs proliferation. RT-PCR and Western blot were used to evaluate gene and protein expression. Beagle dogs were used to establish an animal model of tooth reimplantation and to investigate the effects of scaffolds, BMSCs, SDF-1 and bFGF on periodontal ligament regeneration. X-ray images and micro computed tomography (micro CT) were used to assess morphological changes in replanted teeth and surrounding alveolar bone. H&E staining and Masson’s staining were also performed. BMSCs from Beagle dogs growth on scaffolds consisted of dense structured collagens. SDF-1 and bFGF effectively promoted the differentiation of BMSCs into fibroblasts, periodontal membrane reconstruction, and cell proliferation *in vitro*. SDF-1 and bFGF also stimulated the expression of type I collagen (Col I), type III collagen (Col III), CXC family chemokine receptor 4 (CXCR4), and S100 calcium binding protein A4 (S100A4), and decreased the expression of alkaline phosphatase (ALP). In our experimental Beagle dog model of tooth extraction and replantation, application of SDF-1 and bFGF significantly elevated periodontal membrane reconstruction and thus supported the survival of replanted teeth. In conclusion, the findings from the present study demonstrated that SDF-1 and bFGF enhance the process of periodontal ligament reconstruction, and provide a basis and reference for the use of stem cell tissue engineering in promoting periodontal membrane regeneration.

## Introduction

Tooth regeneration is a serious problem that is difficult to address because of widely recognized difficulties with periodontal ligament regeneration [1]. It is currently believed that periodontal ligament damage compromises the resistance against infection as well as self-renewal and repair. Periodontal ligament cannot be regenerated because tooth dislocation decreases and may even eliminate the function or presence of periodontal ligament cells (PDLCs) [2]. Although the residual periodontal ligament may survive temporarily, the healing is mostly done by fixation. The tooth roots will gradually absorb and be replaced by surrounding bone tissue until they are lost [3]. Therefore, the key to successful periodontal membrane regeneration is maintenance or recovery of active PDLCs [4].

In recent years, new advances in stem cell and tissue engineering research have provided new ideas and possibilities for tooth reimplantation. The ability to repair periodontal defects through periodontal ligament stem cells (PDLSCs) has been confirmed [5,6], but as a future clinical application. Currently, PDLSCs are relatively difficult to use to meet clinical treatment needs. Bone marrow stem cells (BMSCs) are easier to acquire, simpler to culture [7]. Kramer and colleagues [8] demonstrated that BMSCs can differentiate into periodontal ligament fibroblasts after co-culture with PDLCs *in vitro*. Mesenchymal stem cells are the common precursor cells of bone cells and provide a new theoretical basis for the repair of periodontal tissues using BMSCs. Based on these findings, BMSCs may be useful as seed cells for periodontal ligament repair.

Stromal cell derived factor-1 (SDF-1) is a small protein that has chemotactic effects on immune cells. It belongs to the CXC chemokine family and has extensive biological activity. Kim et al. [9] implanted a tooth-shaped biomaterial with SDF-1 in the alveolar fossa of mice in the absence of seed cells, which resulted in regeneration of teeth-like structures. In addition, SDF-1 can stimulate stem cell proliferation and promote type I collagen (Col I) gene expression [10].

Therefore, we investigated the use of SDF-1 together with basic fibroblast growth factor (bFGF) in the tissue engineering of periodontal ligament regeneration, to explore whether it can reduce cell loss, strengthen cell colonization, and stimulate stem cell proliferation on the basis of traditional periodontal tissue engineering. Findings from our study may provide direction for future investigations on mobilizing bone marrow mesenchymal stem cells to target tissues, induction of differentiation into cells with PDLCs characteristics, and mechanisms of action.

## Materials and methods

### Cell culture of BMSCs from beagle dogs

Beagle dogs underwent general anesthesia. The bone marrow was extracted from the tibia (*n*=1, male, 18–24 months, Dashuo Laboratory Animal Co., Ltd., Sichuan, China) by aspiration. All studies involving animals were conducted in accordance with the guidelines set out by the Sichuan University Institute Animal Care and Use Committee. The bone marrow was isolated by gradient density centrifugation and subsequently purified by differential adherence. When cultured primary cells (P0) reached 80–90% confluence (usually 7–10 days), the trypsin digestion solution was added at 37°C for 3–5 min, and the cells were observed under a phase contrast microscope (IX70-S8F2, Olympus Optical, Japan). After cytoplasmic retraction and cell gap increase were observed, complete medium was added to terminate digestion. The first generation (P1) cells were subcultured at a ratio of 1:2. The morphology and growth of the cells were observed under an inverted microscope until the adherent cells were fused and passaged again according to the aforementioned method. Beagle BMSCs were purified by repeated adherence method. Subsequent experiments were performed with P2–P4 cells.

### Flow cytometry assay

The expression of the cell surface markers CD34, CD29, CD44, and CD45 on BMSCs was detected by flow cytometry. P2–P4 Beagle BMSCs were collected, digested, and dissociated into a single cell suspension (5 × 10^6^/ml, 100 μl/tube). The cells were washed with PBS three times, and fixed in 4% paraformaldehyde at 4°C for 15 min after fixation, and washed twice with PBS. Cells were incubated with anti-canine CD29 (SAB4700394, Sigma, U.S.A.), anti-canine CD34 (MCA2411GA Bio-Rad, U.S.A.), anti-canine CD44 (MCA1041G Bio-Rad, U.S.A.)., and anti-canine CD45 (MCA1042A647, Bio-Rad, U.S.A.) antibodies in the dark at room temperature for 30 min. The cells were then washed twice with PBS, resuspended in 100 μl PBS, and then detected by flow cytometry (Beckman Coulter, U.S.A.).

### Osteogenic differentiation and Alizarin Red staining

P2–P4 Beagle BMSCs were digested and resuspended in complete medium (6 × 10^4^ cell/ml). The cell suspension was added to a six-well plate (500 μl/well), to a final volume of 2 ml with complete medium, and cultured at 37°C and 5% CO_2_. After the cells were cultured for 24 h, the medium was carefully discarded and 2 ml of osteogenic induction medium was added to each well. The cells were further incubated at 37°C and 5% CO_2_. Complete medium was used as the control. After 20 days of incubation, cells were washed twice with PBS and fixed in 2 ml of 4% paraformaldehyde for 30 min. After fixation, the cells were washed twice with PBS. Alizarin Red dye solution (1 ml) was added into each well, and the cells were stained for 5 min on a shaker. The cells were washed with PBS two- to three-times and then observed with a phase contrast microscope.

### Adipogenic differentiation and Oil Red O staining

P2–P4 Beagle BMSCs were harvested and resuspended in complete medium (6 × 10^4^ cells/ml). The cell suspension was added to a six-well plate (500 μl/well) to a final volume of 2 ml with complete medium, and the cells were cultured at 37°C and 5% CO_2_. After 72 h, the medium was replaced with 2 ml adipogenic induction medium in each well, and the cells were incubated at 37°C and 5% CO_2_. On post-induction day 14, the induction solution was discarded and cells were washed twice with PBS. The cells were fixed with 2 ml of 4% paraformaldehyde for 30 min. After fixation, cells were washed with PBS two- to three-times and stained with Oil Red O (1 ml) per well. After washing with PBS, the stained cells were observed under an inverted phase contrast microscope.

### Real-time RT-PCR

Gene expression of alkaline phosphatase (ALP), Col I, type III collagen (Col III), CXC family chemokine receptor 4 (CXCR4), and S100 calcium binding protein A4 (S100A4) were evaluated by RT-PCR. P2–P4 Beagle BMSCs were divided into three groups: blank control group (without no growth factor in the medium), SDF-1 group (200 ng/ml SDF-1), and bFGF group (50 ng/ml bFGF). After 5 days of culture, total RNA (50 μl/ group) from the three groups were extracted using TRIzol reagent (cat. no. 15596-018; Invitrogen; Thermo Fisher Scientific, Inc.), cDNA was synthesized using 2 μl total RNA and then reverse transcribed to cDNA by RT assay (DBI Bioscience, Newark, DE, U.S.A.). Subsequently, the relative expression levels of different target genes and controls were analyzed using a SYBR-Green PCR Master Mix kit (cat. no. RR420A; Takara Bio, Inc., Otsu, Japan); the qPCR reactions were performed on an ABI PRISM 7300 Fast Real-Time System (Applied Biosystems; Thermo Fisher Scientific, Inc.). The primer sequences for the genes are listed in Table 1. Cycling conditions included denaturation at 95°C for 30 s, annealing at 95°C for 5 s, and extension at 60°C for 31 s for 45 repeats. This protocol was followed by dissociation at 95°C for 15 s, 60 °C for 1 min, and 95°C for 15 s for one repeat. The experiment was performed three times for each sample. After the PCR amplification reactions were completed, the system automatically plotted the amplification curve and dissolution curve and collected *C*_q_ values. Based on exponential amplification of the target gene and a calibrator, the number of amplified molecules at the quantification cycle was given by 2^−ΔΔ*C*_q_^. The data were assayed with the comparative 2^−ΔΔ*C*_q_^ method to determine the expression levels of target genes.

**Table 1:** The primers used in the experiments

Gene	Primer sequence
*Col I*	Forward: 5′-CTGGAGATAAGGGTGAAGGT-3′
	Reverse: 5′-GGGGGCCTCCTTCACCCTTCTC-3′
*Col III*	Forward: 5′-GAAGCACATCTGGTTTGGAG-3′
	Reverse: 5′-TTGGGGTTGAGGGTTTTACA-3′
*S100A4*	Forward: 5′-CCCAGCTTCTTGGGGAAAAGGA-3′
	Reverse: 5′-GCAGGACAGGAAGACACAGTC-3′
*CXCR4*	Forward: 5′-GAAGAGCTCCATATATACCCTT-3′
	Reverse: 5′-TGGTAACCCATGACCACCATGA-3′
*ALP*	Forward: 5′-CCAAAGGCTTCTTCTTGCTG-3′
	Reverse: 5′-CCACCAAATGTGAAGACGTG-3′
*β-actin*	Forward: 5′-GAAGATCAAGATCATTGCTCCT-3′
	Reverse: 5′-TACTCCTGCTTGCTGATCCACA-3′

### Western blot

Total proteins from the cells were lysed in radioimmunoprecipitation assay buffer (Beyotime, Shanghai, China, cat. no.: P0013B) containing protease inhibitors (BIOSS, Beijing, China). Protein concentration was determined using a BCA Protein Assay kit (Thermo Scientific Pierce, U.S.A., Cat. lot.: 23225). An equal amount of total proteins was then separated by 10% SDS/PAGE and transferred on to a polyvinylidene difluoride membrane (cat. no. PK-NEF1002; PerkinElmer, Inc., Boston, MA, U.S.A.). Next, fat-free milk (5%) was used to block the membranes for 2 h at room temperature. Following blocking, the membranes were incubated with primary antibodies overnight at 4°C. The blots were subsequently incubated with horseradish peroxidase–conjugated secondary antibodies (Wuhan Boster Biological Technology, Ltd., Wuhan, China; cat. no. BA1054) and then developed using enhanced chemiluminescence detection kit (32106, Thermo Scientific™ Pierce, U.S.A.) according to the manufacturer’s protocol. The primary antibodies used included anti-β-actin (4967, CST, U.S.A.), Col I (ab90395, Abcam, U.S.A.), anti-Col III (ab23445, Abcam, U.S.A.), anti-S100A4 (ab41532, Abcam, U.S.A.), anti-CXCR4 (ab219178, Abcam, U.S.A.), and anti-ALP (ab83259, Abcam, U.S.A.) antibodies.

### MTT assay

P2–P4 Beagle BMSCs were digested, collected, and then resuspended in complete medium. Cell density was adjusted to 1 × 10^7^/ml, and the cell suspensions were added to 96-well plates with a micro-loading gun. The wells were inoculated with 2 μl of cell suspension, supplemented with 200 μl medium, and incubated at 37°C and 5% CO_2_. The material-free group was used as the control group, and the collagen membrane material group was the experimental group. The samples were collected at the same time (*n*=10) on days 1, 3, 5, and 7 for methylthiazolyldiphenyl tetrazolium bromide (MTT) colorimetric experiments. After replacing the serum-free medium in each well, 20 μl of filter-sterilized 5 mg/ml MTT solution was added, and the cells were further incubated at 37°C. After hours, the supernatant was carefully discarded, and 150 μl of dimethyl sulfoxide (DMSO) was added at room temperature. The well plates were shaken on a microwell shaker for 10 min to dissolve the crystals. The luminescence absorption optical density (OD) value of each well was measured on a fully automated microplate reader at a wavelength of 570 nm. The cell growth curve was plotted with time as the horizontal axis and optimal density as the vertical axis.

### Surgery

All studies involving animals were conducted in accordance with the guidelines set out by the Sichuan University Institute Animal Care and Use Committee (SKLODLL2013A149). Nine male Beagle dogs (18–24 months old) were used in the experiment. The animal experiments were performed at the Animal Center of Sichuan University. The dogs were anesthetized, and the bilateral mandibular second premolars were removed carefully to avoid injury to alveolar bone and gums. A gelatin sponge was placed into the alveolar sockets for hemostasis. The removed premolars were immediately put into a solution containing two antibiotics. Periodontal membrane in alveolar sockets near the tooth neck and apex of the tooth were removed, and the pulp was scraped off completely. The remaining third of the periodontal membrane in the alveolar sockets at the tooth root was preserved. The gelatin sponge was taken out before replantation, and the teeth were reimplanted, with one side used as the experimental group and the other side as the control group. The extracted teeth immediately underwent root canal treatment, and the periodontal membrane attached to the teeth were scraped off. Treated teeth were washed repeatedly with a solution containing two antibiotics and stored in 75% sterile alcohol. On the first day after surgery, the extracted teeth were washed with PBS, placed in α-MEM medium, and reimplanted into the original alveolar socket. Three groups (three animals per group, and six teeth per group) were set up. In the blank control group (Blank group), the dislocated teeth were directly implanted into the alveolar socket. In the second group (BMSCs group), the dogs’ BMSCs combined with the scaffold material and dislocated teeth were implanted into the alveolar socket. In the third group (SDF-1/bFGF group), the teeth with the dogs’ BMSCs were placed into a solution containing SDF-1 and bFGF (with blank medium used as the control) for 3–4 days and then the teeth combined with the scaffold material were implanted into the alveolar fossa. After implantation in the third group, the intraperitoneal local injection of SDF-1 and bFGF (1 ml, with saline used as control) was performed once every 3 days for 4 weeks. Average looseness of the teeth, bleeding index of the gums, and the height of the periodontal attachment were measured. At the end of the experiments, the animals were killed with carbon monoxide, in accordance with the American Veterinary Medical Association Guidelines for the Euthanasia of Animals 2013 Edition. All animal work was performed at Sichuan University.

### Masson’s staining and H&E staining

The specimens of the oral tissues were removed after the animals were anesthetized with sodium pentobarbital, washed with PBS, and fixed in 10% formalin for 24 h. The specimens were then decalcified in ethylenediaminetetraacetic acid (EDTA) solution for 4 months. Paraffin sections underwent gradient dehydration by incubating the slices in 100% xylene and then in 100, 95, 90, 80, and 70% ethanol. The sections were then placed into xylene for transparentation. The tissue block was placed in an embedding box and embedded in paraffin, from which 7-μm-thick serial slices were prepared. Masson’s staining with Weigert’s iron was performed for 10 min at room temperature according to the manufacturer’s protocol (Gefan biotec., Shanghai, China, cat no.: M029). The stained slices underwent Lichun Red staining for 10 min at room temperature, treated with phosphomolybdic acid, and stained with Aniline Blue. The slices subsequently underwent differentiation in 1% glacial acetic acid for 1 min and were treated with dehydrated 95, 100% alcohol and 100% xylene. The tissues were observed with microscopy (Zeiss AG, Oberkochen, Germany; Axio Observer A1). H&E staining, Masson’s staining, and immunohistochemical images were semi-quantitatively analyzed with Image-Pro® plus 6.0 software (IPP). The proportion of periodontal membrane regeneration and root resorption in each group was calculated. Three viewing fields in each specimen were observed and recorded. The evaluation area was delineated, and the Integrated Optical Density (IOD) was calculated by IPP. The mean optical density (MOD) was calculated as MOD = IOD SUM/Area SUM. For H&E staining, a Hematoxylin and Eosin staining kit (cat. lot.: C0105, Beyotime, China) was used following the manufacturer’s directions.

### Micro CT assay

A complete specimen containing the replanted tooth and its surrounding alveolar bone was selected. The specimen was fixed in a 4% paraformaldehyde solution and placed in a cylindrical micro computed tomography (micro CT) scanning box. The specimen was tightly packed with foam to preserve stability during scanning. During scanning, the long axis of the tooth was kept parallel to the examination bed and perpendicular to the scanning plane in micro CT (Scanco Medical, Bassersdorf, Switzerland). Specimens were scanned from the crown to the root canal, with thickness of the layer set at 10 μm and voltage set at 70 kV. Image analysis software (VGStudio/Max 3D reconstruction software) used the micro CT scan data to reconstruct 3-dimensional images of the teeth and surrounding alveolar bone. Using the 3D reconstructions and micro CT images, the following spatial parameters were obtained: bone volume fraction (BVF), which is the volume of the trabecular bone (bone volume [BV]) divided by the volume of the sample (total volume [TV]); bone surface area volume ratio (bone surface/BV [BS/BV]), which is the surface area of the trabecular bone divided by the volume of the bone; the trabecular numbers (Tb.N); and the trabecular spacing (Tb.Sp). The 5-mm area surrounding the tooth root was selected as the region of interest (ROI) for bone metrology analysis, and analysis of BV/TV, BS/BV, Tb.N, and TB.Sp.

### Statistical analysis

All statistical analyses were performed with SPSS 13.0 software (IBM Corp., Armonk, NY, U.S.A.). The data are presented as mean ± standard error of the mean from three separate experiments. Statistical significance was determined by paired or unpaired Student’s *t* test and one-way analysis of variance (ANOVA) with Tukey’s post-hoc test in cases of standardized expression data. A *P*-value of *P*<0.05 was considered statistically significant. For the micro CT assay, the comparison between the experimental groups and the control group (N) was performed by Student–Newman–Keuls method.

## Results

### Isolation and identification of BMSCs from Beagle dogs and screening of scaffold materials

**Figure 1:**
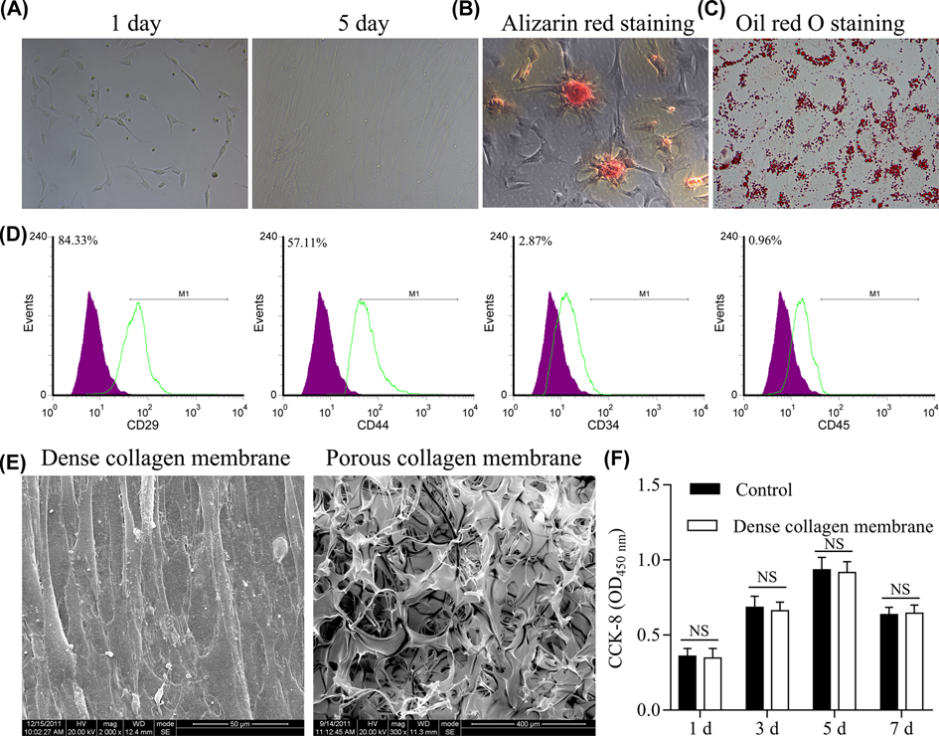
Identification of BMSCs from Beagle dogs and screening of scaffold materials (**A**) A representative image of BMSCs. (**B**) A representative image of Alizarin Red staining of BMSCs. (**C**) A representative image of Oil Red O staining of PBMCs. (**D**) Flow cytometry assay of CD29, CD44, CD4,5 and CD34 on the BMSCs. (**E**) BMSCs cover the surface of dense collagen membrane (left), but not the surface of porous collagen membrane (right). (**F**) Effect of collagen membrane on cell proliferation evaluated by MTT assay.

After the second passage of the primary culture of BMSCs, the P2 cells differentiated into fibroblast-like cells (Figure 1A). Alizarin Red staining revealed that after 20 days of osteogenic induction, P2 BMSCs had formed mineralized nodules, some of which merged into each other (Figure 1B). Oil Red O staining revealed red stained lipid droplets in cells (Figure 1C). Flow cytometry assay of P3 Beagle BMSCs showed the following surface expression profile: CD44^+^ (54.65%), CD29^+^ (86.15%), CD45^−^ (0.77%), and CD34^−^ (2.09%) (Figure 1D).

Electron microscopy assay revealed that dense collagen membrane had good biocompatibility and that cells covered the surface of dense collagen membrane (Figure 1E, **left**). Due to large pore diameter, few cells were found on the porous collagen membrane surface (Figure 1E, **right**). Cell growth was not significantly different between the blank control group and the dense collagen membrane group, as shown by the MTT assay (Figure 1F). These results indicated that the material had good biocompatibility with the cells and no toxic effect.

### Effects of SDF-1 and bFGF on proliferation, differentiation, and chemotaxis of BMSCs *in vitro*

**Figure 2:**
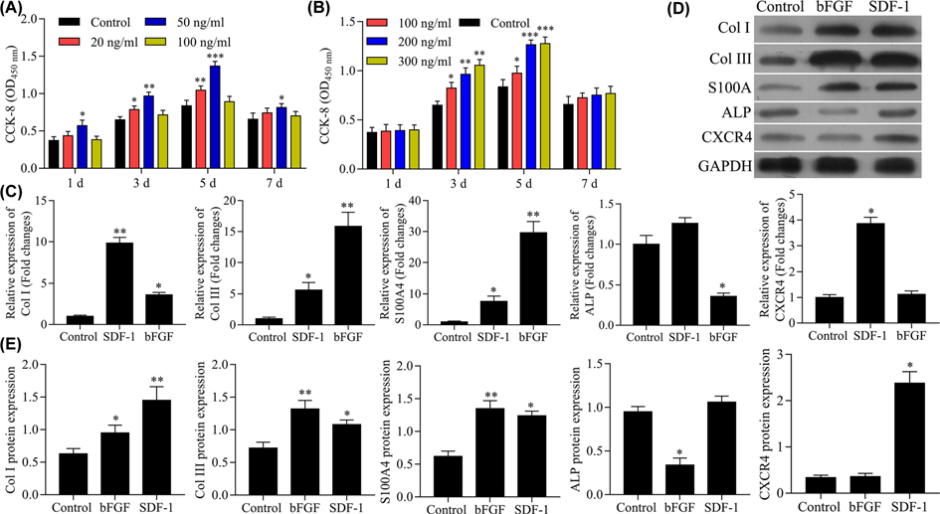
Effects of SDF-1 and bFGF on cell proliferation of BMSCs from Beagle dogs and screening of scaffold materials (**A**) Effects of SDF-1 on cell proliferation evaluated by MTT assay. (**B**) Effects of bFGF on cell proliferation evaluated by MTT assay. (**C**) Effects of SDF-1 and bFGF on gene expression of Col I, Col III, ALP, S100A4, and CXCR4 evaluated by RT-PCR. (**D,E**) Effects of SDF-1 and bFGF on gene expression of Col I, Col III, ALP, S100A4, and CXCR4 revaluated by Western blot. ***P<0.001, **P<0.01, *P<0.05, *vs.* control group.

As shown in Figure 2A,B, bFGF (20, 50, and 100 ng/ml) or SDF-1 (100, 200, and 300 ng/ml) could both promote cell proliferation of BMSCs. bFGF (50 ng/ml) and SDF-1 (200 and 300 ng/ml) had optimal pro-proliferating effects. Therefore, the combination of bFGF (50 ng/ml) and SDF-1 (200 ng/ml) was used in subsequent experiments.

We investigated the effects of SDF-1 and bFGF on gene expression and protein levels of Col I, Col III, S100A4, ALP, and CXCR4. RT-PCR assay showed that gene expression of Col I and Col III, and S100A4 were significantly increased by both SDF-1 and bFGF; the increase in Col I gene expression was greater with SDF-1 compared with bFGF, while the increase in Col III and S100A4 gene expression was greater than bFGF compared with SDF-1 (Figure 2C). The gene expression of ALP was inhibited in bFGF-treated cells but was not affected inSDF-1-treated cells (Figure 2C). The expression of CXCR4 was increased in SDF-1-treated cells but not in cells bFGF-treated cells (Figure 2C).

Results of Western blot confirmed the findings of the RT-PCR assay. Both SDF-1 and bFGF increased the expression of Col I, Col III, and S100A4, but Col I levels were higher in the SDF-1 group vs the bFGF group, and relatively higher levels of Col III and S100A4 were found in the bFGF group vs the SDF-1 group (Figure 2D,E). ALP protein levels were lower in the bFGF group vs the SDF-1 group, which had no significant effect on the protein level of ALP (Figure 2D,E). The secretion of CXCR4 protein from BSMCs was significantly increased only in the SDF-1 group (Figure 2D,E). These results indicated that the combination of SDF-1 and bFGF produced more beneficial effects in BMSCs.

### SDF-1 and bFGF promote BMSCs-induced periodontal ligament reconstruction in Beagle dogs

**Figure 3:**
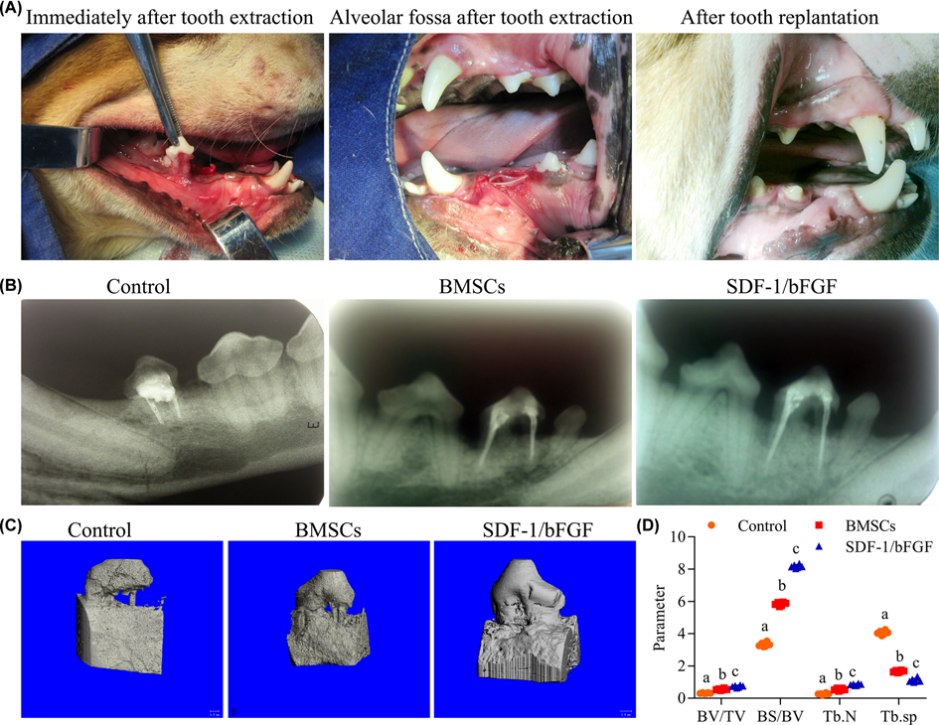
Morphological changes induced by SDF-1 and bFGF combined with BMSCs and biomaterial in tooth reimplantation in Beagle dogs as assessed by X-ray and micro-CT (**A**) Photo of surgery. The second premolar of the lower jaw was removed (left). Alveolar fossa after tooth extraction, with the collagen membrane implanted in the alveolar socket (middle). Three months after teeth replantation, the teeth grew well (arrows point to replantation) and there was no acute inflammation of the periodontal tissue (right). (**B**) X-ray picture of reimplanted teeth in the Blank, BMSCs, and SDF-1/bFGF groups after teeth replantation. No periodontal ligament gap was observed in the control group, but periodontal ligament was visible in the proximal middle root in the BMSCs group (left). A black periodontal ligament gap was visible in the SDF-1/bFGF group (right). (**C**) Representative images of the 3-dimensional structure of reimplantation tooth tissues. Complete absorption of one of the root surface and the bone tissue around the root in the control group. Partial absorption of the root in the BMSCs group. Continuous tooth root and minimal absorption of alveolar bone in the SDF-1/bFGF group. (**D**) BV/TV, BS/BV, Tb.N and Tb.sp in the Blank, BMSCs, and SDF-1/bFGF groups (*n*=6). Different letters mean significant difference in different groups.

Morphology studies of X-ray and micro CT revealed that SDF-1 and bFGF combined with BMSCs and biomaterial were effective in promoting tooth reconstruction. Periodontal membrane regeneration is critical for tooth preimplantation and is evident as a periodontal ligament gap approximately one-third of the root crown. The animal model for periodontal ligament regeneration was established (Figure 3A). In X-ray assay, the roots of reimplanted teeth with no disposal were directly adhered to the alveolar bone, and no periodontal membrane structure could be found (Figure 3B, **left**). In the BMSCs group, the periodontal ligament gap was found (Figure 3B, **middle**). In animals treated with the SDF-1/bFGF combination, a more pronounced periodontal ligament gap was found, indicating regeneration of the periodontal membrane (Figure 3B, **right**).

Micro CT three-dimensional image reconstructions showed obvious bone resorption of tooth root that was more obvious in the Blank group than in the BMSCs group and SDF-1/bFGF group (Figure 3C, left). Partial absorption of tooth root and alveolar bone was found in the BMSCs group (Figure 3C, middle). Minimal bone resorption of tooth root and alveolar bone was observed in the SDF-1/bFGF group (Figure 3C, right). Image analysis of the micro CT assay revealed that among the three groups, the SDF-1/bFGF group had the highest periodontal membrane healing rate and lowest root resorption rate.

Quantitative results of BV/TV, BS/BV, Tb.N, and Tb.Sp from ROI area analysis in the micro CT assay provided additional data on the effects of SDF-1/bFGF on tooth reconstruction (Figure 3D). BV/TV was evaluated; a decrease in BV/TV indicates bone reabsorption. Among the three groups, the smallest BV/TV was found in control group. Both BMSCs and SDF-1/bFGF treatment promoted BV/TV, but animals in the SDF-1/bFGF group had the highest BV/TV. The spatial parameter BS/BV indicates the amount of trabecular bone surface area as a proportion of total bone surface area and was significantly higher (i.e., had lower bone absorption) in the SDF-1/bFGF group than in the other two groups. The special parameters Tb.N and Tb.Sp, which indicate the number and dispersion of trabecular bone, increased in the SDF-1/bFGF group, suggesting partial reconstruction of the alveolar bone tissue. Tb.N of the ROI area in the SDF-1/bFGF group was relatively higher than in the Blank and BMSCs groups, while Tb.Sp which indicated dispersion of trabecular bone, was minimal in the SDF-1/bFGF group compared with the other two groups.

**Figure 4:**
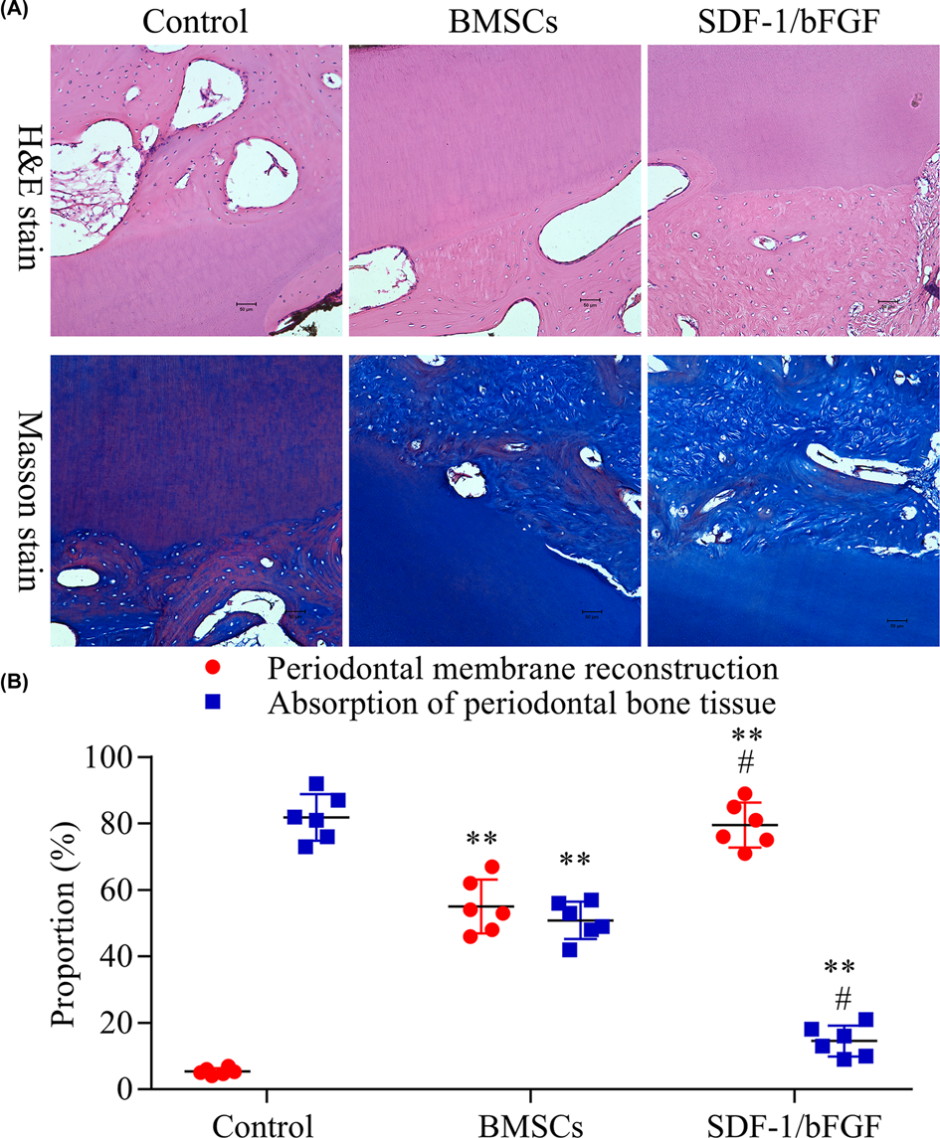
H&E staining and Masson’s staining of SDF-1 and bFGF promote BMSCs-induced periodontal ligament reconstruction in Beagle dogs (**A**) Representative H&E and Masson’s staining images of the control, BMSCs and SDF-1/bFGF groups after teeth replantation. (**B**) Root group absorption ratio of different groups (*n*=6). One-way ANOVA, **P<0.01 *vs.* Blank group, ^#^*P*<0.05 *vs.* BMSCs group.

No acute inflammatory response was observed in any of the stained tissue slices. The results of Masson’s staining and H&E staining were consistent. Compared with the Blank group, periodontal membrane reconstruction was observed in both the BMSCs group and SDF-1/bFGF group, and absorption of periodontal bone tissue was relieved by the treatment. The original periodontal membrane fibers in the Blank group disappeared, and no new periodontal ligament fibers were found. In the BMSCs group, new periodontal ligament fibers were observed, and most of the fibers were disordered, partially inserted obliquely or vertically into the tooth root surface. In the SDF-1/bFGF group, the fibers around tooth root were more ordered, with one end inserted obliquely into the surface of the root and the other end buried in the alveolar bone (Figure 4).

## Discussion

The periodontal ligament is critical in maintaining normal tooth function and prevents bone and cement adhesion during periodontal tissue regeneration. The periodontal membrane maintains physiological conditions that protect PDLCs from mineralization [11]. A regulatory mechanism in the periodontal ligament may inhibit osteoblast differentiation, maintain and balance the periodontal ligament fibroblast phenotype, and control the degree of osteogenesis in bone remodeling. Our study demonstrated that SDF-1/bFGF can promote the survival of reimplanted teeth by regulating the process of osteoblast differentiation and stimulating the expression of Col I, Col III, and S100A4.

SDF-1, which is also known as CXCL12, was first discovered as cytokine and was later identified as a member of the chemokine CXC subfamily [12]. SDF-1 is expressed ubiquitously [13–16], and its receptor CXCR4 is widely expressed on the surface of leukocytes, CD34^+^ hematopoietic stem cells, and CD34^+^ progenitors [17]. SDF-1 levels are elevated at 5 days post-injury at the site of the wound, suggesting late-phase expression of SDF-1 during repair [18]. In an animal model of vagotomy, injection of SDF-1α into the intracranial nerve injury site promoted differentiation of nerve cells [19]. Furthermore, a rise in the local expression of SDF-1 can promote the migration of BMSCs to the site of injury after systemic injection [20]. These findings suggest that SDF-1 regulates the migration and differentiation of stem cells to target tissues. bFGF plays an important role in cell growth, proliferation, differentiation, but also has a role in other cellular functions [21–23]. For example, bFGF not only improves the proliferation rate and longevity of BMSCs, but also maintains the multi-differentiation potential of BMSCs during proliferation [23,24] and promotes adherence of BMSCs [25,26].

We have demonstrated that SDF-1 and bFGF can stimulate the expression of Col I, Col III, and S100A4. Others have shown that S100A4 and Col III inhibit mineralization and may stabilize the structure and function of periodontal membrane [27,28]. Type III collagen accounts for 11.73% of the periodontal ligament and is a biomarker of tissue repair [29,30]. S100A4 is a member of the S100 calcium-binding protein family, which is synthesized and secreted by PDLCs [31]. Recombinant murine S100A4 protein inhibited mineralization in bone marrow cells in a concentration-dependent manner. Duarte et al. [31] suggested that S100A4 may also participate in the response of PDLC to mechanical stress. Kato et al. reported that inhibition of human periodontal ligament S100A4 promotes the expression of osteoblast markers [32], while application of S100A4 inhibits osteoblast gene expression and mineralization in the periodontal ligament. Type III collagen and S100A4 can prevent the deposition of mineralized matrix, which may preserve the normal gap in periodontal ligament without mineralization [33]. In our study, SDF-1 and bFGF stimulated the differentiation of BMSCs into periodontal ligament fibroblasts and increased the expression of related genes and proteins that inhibit mineralization. Higher ALP expression, indicative of calcium salt deposition, has also been reported. However, bFGF supports periodontal membrane regeneration by inhibiting the expression of ALP [34]. The results of our *in vitro* studies are consistent with previous studies. Additionally, the expression of CXCR4 was shown to be significantly increased in BMSCs treated with SDF-1. Increased expression of CXCR4, which is the only chemotactic receptor for SDF-1, is would likely enhance the chemotaxis of SDF-1. Increased CXCR4 expression also activates exogenous SDF-1, which in turn enhances cell migration and chemotaxis.

In our *in vivo* animal experiments, the combination of SDF-1/bFGF, BMSCs, and scaffold promoted the survival of reimplanted tooth. This finding was consistent data from a previous study showing that bFGF that can stimulate local cementation [35]. Gibran et al. [36] and Murakami et al. [37] reported that topical application of bFGF can stimulate cell differentiation in periodontal residual tissues in the early stage of trauma and affect the regeneration of surrounding periodontal tissues. In our study, treatment with bFGF and SDF-1 promoted pronounced healing of the periodontal membrane, and formation of new cementum on the root surface. Additionally, inflammatory cell infiltration was reduced in SDF-1 and bFGF treated samples. We speculate that SDF-1 recruits not only BMSCs to the lesion area, but also host defense cells that support wound healing and tissue repair and may be involved in immune surveillance. The inflammatory response to biological materials is mediated by mast cell activity [38], and CXCR4-positive mast cells affect the concentration gradient of SDF-1 *in vitro* [39]. These previous studies suggested a mechanism by which SDF-1 may regulate the inflammatory response. The homing effect of stem cells and the influence of cytokines on the tissue engineering microenvironment are potential important areas for future study in stem cell tissue engineering.

In summary, regeneration of the periodontal ligament is a complicated process involving chemotaxis of stem cells to specific sites and regulation of the microenvironment of stem cell. The results of our study provide a preliminary theoretical basis and reference for the use of chemokine SDF-1 and cytokine bFGF to promote BMSC-mediated periodontal membrane regeneration and lay a foundation for subsequent in-depth research.

## Abbreviations

ALP: alkaline phosphatase
bFGF: basic fibroblast growth factor
BMSC: bone marrow stem cell
BV: bone volume
Col I: type I collagen
Col III: type III collagen
CXCR4: CXC family chemokine receptor 4
IOD: integrated optical density
IPP: Image-Pro® plus 6.0 software
micro CT: micro computed tomography
MOD: mean optical density
MTT: methylthiazolyldiphenyl tetrazolium bromide
PDLC: periodontal ligament cell
PDLSC: periodontal ligament stem cell
ROI: region of interest
SDF-1: stromal cell derived factor-1
S100A4: S100 calcium binding protein A4
Tb.N: trabecular number
Tb.Sp: trabecular spacing
TV: total volume
